# Outcomes and factors affecting mortality and successful tracing among patients lost to follow-up from antiretroviral therapy in Pawi Hospital, Northwest Ethiopia

**DOI:** 10.1186/s41182-019-0181-6

**Published:** 2019-11-06

**Authors:** Moges Agazhe Assemie, Cheru Tesema Leshargie, Pammla Petrucka

**Affiliations:** 1 Department of Public Health, College of Health Science, Debre Markos University, P.O. Box 269, Debre Markos, Ethiopia; 2 Department of Environmental Health, College of Health Science, Debre Markos University, Debre Markos, Ethiopia; 3 College of Nursing, University of Saskatchewan, Saskatoon, Canada; 4 School of Life Sciences and Bioengineering, Nelson Mandela African Institute of Science and Technology, Arusha, Tanzania

**Keywords:** Antiretroviral therapy, Loss to follow-up, Mortality, ART tracing, Cotrimoxazole preventive therapy, Ethiopia

## Abstract

**Background:**

Loss to follow-up (LTFU) is a major public health problem to antiretroviral therapy (ART) programs in sub-Saharan Africa. Failure to account for patients’ LTFU outcomes (self-transfers and restarts) can result in inaccurate reporting of retention in care. In Ethiopia, specifically in the Benishangule Gumuz region, high LTFU reported patients, who were not assessed for their outcomes, are identified as a gap. Therefore, our objective was to determine the outcomes (alive or dead) of patients lost to follow-up (LTFU) from ART and identify factors associated with successful tracing and mortality of these patients.

**Results:**

The proportion of successful tracing was 75.5% (249 of 330). Among the traced patients (*n* = 249), 22.9% were deceased, 47.8% were on ART, and 29.3% had discontinued treatment. However, the remaining untraceable patients were not locatable due to wrong addresses (53.1%), change of residence (29.6%), and/or lack of functional phone contact (17.3%). Some (32.9%) of the patients discontinued because of negative test results, others (21.9%) for spiritual reasons or side effects (28.8%), and the remaining (16.4%) for other reasons. Tracing using phone numbers (AOR = 2.97, 95% CI 1.57–5.59) and existing long-term follow-up period for ART (AOR = 2.13, 95% CI 1.17–3.88) were strong predictors of successful tracing while not receiving cotrimoxazole preventive therapy (CPT) (AOR = 2.59, 95% CI 1.22–5.39) is a predictor for mortality of patients post-LTFU.

**Conclusion:**

ART programs need to retain current contact information of patients or guardians/friends for tracing. Having phone contact numbers and prolonged lengths of compliance with ART are predictors of successful tracing, while lack of cotrimoxazole preventive therapy is a predictor of mortality. Early tracing of beginners (newly admitted recipients) and updating their detailed information at each follow-up visit is essential.

## Background

Highly active antiretroviral therapy (HAART) has become widely available in low- and middle-income countries where human immunodeficiency virus (HIV) infection and acquired immune deficiency syndrome (AIDS) were most prevalent. By the end of 2015, 17 million people globally had received ART, while at least 12 million people were living with HIV in sub-Saharan Africa [1].

Retention in care and support are important measures of ART program effectiveness and critical in achieving the 90-90-90 targets (90% of people living with human immunodeficiency virus (PLHIV) know their status, 90% of PLHIV who know their status are on treatment (ART), and 90% of PLHIV on treatment have attained viral suppression) [1–3]. “Loss to follow-up” is a general term for unknown outcomes of patients who have interrupted or not attend their ART medication follow-up visits. Studies in Ethiopia indicated a high proportion of loss to follow-up (LTFU), ranging between 14.5% and 22.6% for ART [4–8]. Outcomes for LTFU patients were classified into four categories: self-transfer, restart, discontinuance, and death [9, 10]. Therefore, it is possible to determine the actual outcomes of LTFU through active tracing by phone calls and field/home visits [11–13].

However, outcomes of post-LTFU patients have not been identified or described in the literature. Failure to account for patients’ outcomes (self-transfer and restart) can result in inaccurate reporting of retention in care, which is an essential indicator of measures of the success of ART scale-up as well as ensuring supply chain management of laboratory investigations, medications, and human resource requirements [14].

In Ethiopia, LTFU patients are not assessed for their outcomes; hence, this study aimed to determine outcomes of patients LTFU and identify factors associated with tracing and mortality of these patients. By assessing outcomes and factors affecting mortality and tracing will provide information for clinicians seeking appropriate management of patients on HAART. Moreover, policy makers and public health professionals will use the findings to design ART-related policies, programs, and services.

## Methods and materials

We conducted a cross-sectional study at Pawi General Hospital (PGH). PGH is one of three (Assossa, Pawi, and Kamashi) hospital clinics that provide ART services in Benishangule Gumuz Region and is found in the Metekel zone, Northwest Ethiopia. The hospital has provided chronic HIV care and support to both pre-ART and ART clients since 2005. Between April 2007 and March 2017, 1760 patients were started on ART at this site.

The study population included all adult patients age 18 and older, who started ART at PGH and classified as LTFU. Identified LTFU patients and their baseline information were extracted from hospital records/charts retrospectively from 2007 to 2017. Lost to follow-up was considered for those who were not taking an ART refill for a period of 3 months and above. Finally, tracing was conducted from April 1 to June 30, 2017, and prevalence of tracing was determined at a given LTFU population. To check sample adequacy, a minimum sample size of 296 was determined based on single population proportion formula with consideration of a 10% non-response rate, and finally we surveyed all 330 patients in the given hospital.

When patients start ART, their baseline characteristics are entered on master cards, which are required at each monthly visit to the ART clinic to collect the next month’s supply of ART drugs. If a patient is not seen in the clinic for three consecutive months, the patient’s status is termed as “lost to follow-up” on the master card and the register book. We identified all adult patients listed as LTFU on the master cards and registers. We excluded patients whose contact address was outside Metekel zone, Benishangule Gumuz, Ethiopia and/or whose phone contact was not provided.

The operational definitions of LTFU was those who were not taking an ART refill for a period of 3 months or longer from the last attendance for refill. Transferred out reflected those patients who were formally transferred out to another health facility. Working functional status is defined as those patients who were able to perform their day-to-day activities. Negative test result refers to those patients who had started ART with a false positive test and then become negative or a negative test result with a false negative diagnosis but may actually be positive.

Data quality was maintained by a pre-tested data collection tool to collect relevant information on LTFU outcomes of current ART status and capturing LTFU elements such as restart at former clinic, self-transfer to other clinics discontinued, or deceased. Training was given for HIV-positive outreach tracers who visited patient homes and made phone calls between April 1 and June 30, 2017. Close supervision and verifications of data completeness were done. If patients were traced directly, they were asked whether they were still taking ART and, if not, why they discontinued. If a patient had died or moved away, the relatives were asked when the patient died or moved which was also entered into the data set.

### Data management and statistical analysis

We used descriptive analysis to examine demographic and clinical characteristics as well as the history of LTFU patients and their outcomes. Outcomes were classified as alive, dead, or untraceable. Patients found alive were further categorized as restart, self-transferred out to another clinic, or discontinued. Logistic regression model was fitted to determine associated factors, and variables with *p* value below 0.25 in bivariate analysis were entered to multivariable analysis to adjust odds ratios that were mutually adjusted for all other predictor variables. The adjusted odds ratios with a *p* value ≤ 0.05 were considered statistically significant, and model fitness was assessed by Hosmer-Lemeshow goodness of fit test for logistic regression. The data was entered by Epi Info 7, and further analyses were done by using Stata/se Version 14 software.

## Results

There were 480 (27%) patients classified as LTFU from the pool of 1760 ART-initiated HIV-positive patients at Pawi hospital in the study period.

### Socio-demographic characteristics of LTFU patients

Of 330 patients eligible for tracing, 54.5% were female. The mean age of the study participants was 33.3 (range 32.1 to 34.4) years. One quarter (62; 25%) of the patients participating in the study had secondary or higher education, with 35.7% lacking any formal education. Marital status of the study participants showed 98 (39.4%) were married, while 56 (22.5%) of the patients were divorced. One in seven (39; 15.7%) of the patients were government employees while 94 (37.7%) were farmers (Table 1).

**Table 1:** Socio-demographic characteristics of patients included in the study

Variables	Numbers (%)
Sex of respondent	
Male	150 (45.5)
Female	180 (54.5)
Age	
18–28 years	119 (36.1)
29–40 years	136 (41.2)
> 40 years	75 (23.7)
Marital status	
Never married	72 (28.9)
Married	98 (39.4)
Divorced	56 (22.5)
Widowed	23 (9.2)
Educational status	
No education	89 (35.7)
Primary	98 (39.3)
Secondary and above	62 (25.0)
Occupation	
Government employee	39 (15.7)
Daily laborer	72 (28.9)
Farmer	94 (37.7)
Self-employed	44 (17.7)

### Baseline clinical and related characteristics of LTFU patients

The mean duration of follow-up time on ART clinic before being LTFU was 16.47 months (range 13.7 to 19.3) and two thirds (222; 67.3%) of the patients were on ART for at least 12 months prior to LTFU. The mean body mass indexes of the patients were 19.3 found in the normal range of 18.5 to 24.9 kg/m^2^. The median baseline CD4 cell count/μl was 182 (IQR 92–271). The number of study participants receiving cotrimoxazole preventive therapy (CPT) was 211 (64%). However, 131 (39.7%) of the patients had developed opportunistic infection. Approximately two fifth 124 (37.6%) of the patients successfully traced had baseline working functional status. Conversely, 226 (68.5%) of the patients were in an advanced clinical stage (III/IV) before LTFU (Table 2).

**Table 2:** Baseline clinical and related characteristics of patients included in the study

Variable	Number (%)
Functional status	
Working	124 (37.6)
Ambulatory	91 (28.6)
Bedridden	115 (34.8)
CPT	
Received	211 (64)
Not received	119 (36)
Isoniazid preventive therapy (IPT)	
Not received IPT	235 (71.21)
Received IPT	95 (28.79)
Duration of follow-up on ART	
Follow-up time < 12 months	222 (67)
Follow-up time > 12 months	108 (33)
WHO clinical stage	
I	35 (11)
II	69 (21)
III	140 (42)
IV	86 (26)
Opportunistic infection	
Opportunistic infection	131 (39.70)
No opportunistic infection	199 (60.30)

### Successful tracing of LTFU patients

Three hundred and thirty of the LTFU patients were eligible for tracing of which 249 (75.45%) of the patients were successfully traced. Of these located LTFU patients, the status of 98 (39.4%) patients found at hospital traced records and the remaining 151 (60.6%) had to be traced by outreach. Tracing by field/home visit was done for 220 (66.7%) patients with 154 (61.8%) being located, while tracing via phone contact was undertaken for 110 (33.3%) with a successful contact with 95 (38.2%) patients.

### Outcomes of LTFU patients

From the 249 LTFU patients who were successfully traced and evaluated for their LTFU outcomes, 192 (77.1%) were alive and 57 (22.9%) dead. Of those who were alive, 79 (41.2%) were on ART at Pawi Hospital, 40 (20.8%) at another ART clinic, and 73 (38%) had stopped ART. For 16 (21.9%) of those who stopped treatment, the rationale was due to a spiritual belief; for 21 (28.8%), side effects were the primary reason for discontinuance; 24 (32.9%) believed they were cured (negative test); and for 12 (16.4%), no reasons were specified. One fourth (24.5%) of the study population was not traced successfully due to wrong address, 43 (53.1%); change of residence, 24 (29.6%); and/or phone contact failure (17.3%).

### Factors associated with successful tracing of LTFU patients

All the variables (i.e., CPT, age category, tracing methods, follow-up period, WHO clinical stage, sex, occupation, marital status, educational status, opportunistic infection, and isoniazid preventive therapy) were tested in the bivariable analysis. Those variables with a *p* value below 0.25 were entered into multivariable logistic regression analysis. Accordingly, CPT, age category, tracing methods, follow-up period, and WHO clinical stage were entered to the final analysis, and we found that persons who had at least 12 months of ART and then left were more likely to be traced (AOR = 2.13, 95% CI 1.17–3.88, *p* = 0.013) and having a phone number with the odds of successful tracing (AOR = 2.71, 95% CI 1.47,5.03, *p* = 0.001) was a statistically significant factor for successful tracing by controlling all other variables (Table 3).

**Table 3:** Factors associated with successful tracing of LTFU adult HIV-positive patients

Variable	Tracing *N* (%)		Crude OR (95% CI)	Adjusted OR (95% CI)	*p* value
	Not traced	Traced			
Age					
18–28 years	27 (33.3)	92 (37)	1	1	
29–40 years	35 (43.2)	101 (41)	0.84 (0.47,1.51)	0.88 (0.49–1.60)	0.674
> 40 years	19 (23.5)	56 (22)	0.86 (0.44,1.70)	0.93 (0.46–1.87)	0.842
CPT					
Not received	28 (34.6)	91 (37)	1.09 (0.64,1.84)	0.89 (0.51–1.56)	0.679
Received	53 (65.4)	158 (63)	1	1	
Tracing methods					
Phone	15 (18.5)	95 (38)	2.71 (1.47,5.03)	2.97 (1.58–5.59)	0.001*
Field/home visit	66 (81.5)	154 (62)	1	1	
Clinical stage					
I/II	24 (29.6)	80 (32)	1	1	
III	33 (40.7)	107 (43)	0.97 (0.53,1.77)	1.09 (0.59–2.02)	0.793
IV	24 (29.6)	62 (25)	0.78 (0.40,5.26)	0.72 (0.36–1.45)	0.364
Follow-up time					
< 12 months	62 (76.5)	160 (64)	1	1	
> 12 months	19 (23.5)	89 (36)	1.82 (1.02,3.23)	2.13 (1.17–3.88)	0.013*

*p value for statistically significant variable

### Factors associated with mortality of LTFU patients

Based on bivariable logistic regressions analysis, CPT, age category, tracing methods, follow-up period, and WHO clinical stage were candidates for multivariable logistic regression with *p* values below 0.25. In multivariable logistic regressions, CPT was found the only statistically significant factor for mortality with the odds of AOR 2.98, 95% CI 1.46–6.12, *p* = 0.013, by controlling for all other variables (Table 4).

**Table 4:** Factors associated with mortality of adult HIV-positive patients LTFU

Variable	Outcomes number (%)		Crude OR (95% CI)	Adjusted OR (95% CI)	*p* value
	Deceased	Alive			
Age category					
18–28 years	17 (29.8)	75 (39.1)	1	1	
29–40 years	23 (40.4)	78 (40.6)	0.77 (0.38,1.55)	0.78 (0.38–1.60)	0.491
> 40 years	17 (29.8)	39 (20.3)	0.52 (0.24,1.13)	0.54 (0.24–1.20)	0.129
CPT					
Not received	11 (19.3)	80 (41.7)	2.98 (1.46,6.12)	2.59 (1.22–5.59)	0.013*
Received	46 (80.7)	112 (58.3)	1	1	1
Tracing methods					
Phone	16 (28.1)	79 (41.1)	1.79 (0.94,3.42)	1.53 (0.78–3.02)	0.217
Field work	41 (71.9)	113 (58.9)	1	1	1
Clinical stage					
I/II	16 (28.1)	64 (33.3)	1	1	
III	22 (38.6)	85 (44.3)	0.97 (0.45,1.99)	1.03 (0.49–2.17)	0.932
IV	19 (33.3)	43 (22.4)	0.57 (0.26,1.22)	0.69 (0.31–1.54)	0.369
Follow-up time					
< 12 months	40 (70.2)	120 (62.5)	1	1	
> 12 months	17 (29.8)	72 (37.5)	1.41 (0.75,2.67)	1.57 (0.77–2.94)	0.231

*p value for statistically significant variable

## Discussion

Exhaustive tracing studies of LTFU patients are important efforts to improve the quality of care as well as the outcome evaluation of ART programs in Ethiopia. Hence, this study was conducted to determine outcomes and factors affecting mortality and successful tracing among patients LTFU from ART. We achieved 75.5% successful tracing for LTFU patients which revealed mortality of 22.9%. In addition to deceased, live patients further identified as LTFU causes of 20.8% were self-transferred to other ART clinics, 41.2% were on ART at Pawi Hospital, and 38% had discontinued the treatment. Moreover, having a phone number and completing follow-up for more than a year were significantly associated with successful tracing whereas CPT was the only significant predictor of mortality.

The proportion of successful tracing was in line with previous studies conducted in Kenya [15] and Malawi [16] and a systematic review of studies in Africa [17]. However, the findings in this study are higher than those of a systematic review in other resource-limited settings [18] which could reflect that most of the residents in the study area are difficult to trace due to issues in addressing. In our study, tracing via phone contacts and longer follow-up on ART were more successful in revealing the outcomes of LTFU. But, as in other studies, we failed to find any significant relations among age categories, baseline advanced clinical stage, functional status, CPT, and baseline CD4 cell count with tracing success [13, 16].

The availability of phone contacts appears an effective way of identifying outcomes of LTFU patients. Via telephone, patients were more likely to be located on a first attempt, to admit leaving the clinic, and to reveal their outcome status. Likewise, LTFU survivors are less likely to change their personal or friends’ phone numbers leading to successful phone tracing. Both scenarios could decrease the proportion of tracing patients via in-person field tracing compared to phone calls as observed in our study and mirrored in a study in Malawi [16]. Cell phone coverage is increasing the positive experience of using phones in patient follow-up, resulting in more successful tracing [7]. However, this study conflicts with findings from a studies in South Africa [17, 19], although its confidence interval was very wide (95% CI 1.5–47.7), indicating a large variability and weak evidence.

Longer length of time attending ART before stopping was a predictor for successful tracing. Longer length of time attending ART may increase patients’ familiarity with all service providers in terms the details of their personal biography, which minimizes incomplete patients’ contact details.

This study also indicated that 23% of the patients traced had died, which is similar to the findings in a Kenyan study [15] which could be due to relatively similar settings in Ethiopia. However, it is lower than studies in Malawi and other select resource-limited settings of sub-Saharan Africa [12, 17, 20], which may be attributable to variations in study settings and periods.

These findings suggest that CPT is a strong predictor of mortality among traced patients that missed scheduled visit dates. Adults who did not receive CPT had significantly higher incidences of mortality as end points. This finding could be due to LTFU patients also choosing to discontinue CPT despite its potential to reduce mortality by reducing comorbidities during the LTFU period [9, 18, 21, 22]. In our study, advanced age, advanced WHO stages, and lower baseline CD4 counts were not significantly predictive for patients who died.

### Limitation of the study

One limitation of this work is excluding patients who have no phone contact and home address outside of the Metekel zone due to lack of resources and technology. In addition, dependence on patient’s or family/friends’ recall of the details of the ART services they have received, or their tendency to report outcomes as they feel desirable or acceptable to the researchers/tracers is also a limitation. This inaccurate/misgiven information could bias reporting of the outcomes of patients LTFU.

## Conclusions

ART programs need to retain current contact information of patients or family/friends for tracing. Having phone contact numbers and prolonged lengths of compliance with ART are predictors of successful tracing, while lack of CPT is a predictor of mortality. Early tracing of beginners (newly admitted recipients) and updating their detailed information at each follow-up visit is essential.

## Data Availability

The datasets used and/or analyzed during this study are available from the corresponding author (MAA) on reasonable request.

## Abbreviations

AOR: Adjusted odds ratio
ART: Antiretroviral therapy
CI: Confidence interval
CPT: Cotrimoxazole preventive therapy
HAART: Highly active antiretroviral therapy
LTFU: Loss to follow-up
PGH: Pawi General Hospital
PLHIV: People living with human immunodeficiency virus
WHO: World Health Organization
